# Evaluation of the Antimicrobial Activity of Bimetallic TiO_2_-ZnO Nanoparticles Phytosynthesized with *Ruta graveolens* Extract Supported on a Chitosan Film

**DOI:** 10.3390/polym18040447

**Published:** 2026-02-10

**Authors:** Angelica Monserrat Velázquez-Hernández, Sonia Martínez-Gallegos, Julio César González-Juárez, Julio Villalobos-Brito, Verónica Albiter, Martha Manjarrez-Olvera, Carlos García-Ibarra, Javier Illescas

**Affiliations:** 1 Tecnológico Nacional de México/Instituto Tecnológico de Morelia, División de Estudios de Posgrado e Investigación, Av. Tecnológico 1500, Lomas de Santiaguito, Morelia CP 58120, Mexico; julio.gj@morelia.tecnm.mx (J.C.G.-J.); julio.vb@morelia.tecnm.mx (J.V.-B.); 2 Tecnológico Nacional de México/Instituto Tecnológico de Toluca, División de Estudios de Posgrado e Investigación, Av. Tecnológico S/N, Agrícola Bellavista, Metepec CP 52149, Mexico; malbiterl@toluca.tecnm.mx (V.A.); mmanjarrezo@toluca.tecnm.mx (M.M.-O.); cagi@gmail.com (C.G.-I.); fillescasm@toluca.tecnm.mx (J.I.)

**Keywords:** chitosan, nanoparticles, TiO_2_-ZnO, phytosynthesis, *Ruta graveolens*

## Abstract

In this work, bimetallic TiO_2_-ZnO nanoparticles were phytosynthesized in molar ratios of 1Ti:1Zn, 2Ti:1Zn, and 1Ti:2Zn, using plant extract of *Ruta graveolens* leaves as reductant agent. The TiO_2_-ZnO nanoparticles were supported on chitosan films at concentrations of 0.1 and 0.2% (*w*/*v*). The resulting films were characterized by FTIR, TGA-DT and DSC analysis, confirming an adequate impregnation of the nanoparticles in the polymeric matrix and stability against temperature variations, while swelling tests revealed good structural strength without appreciable deformation. The antimicrobial activity of the membranes was evaluated by the disk diffusion method (Kirby–Bauer) against *Escherichia coli*, *Staphylococcus aureus* and *Candida albicans*. It was observed that the membranes having nanoparticles of stoichiometric ratios 1Ti:1Zn and 2Ti:1Zn presented higher microbicidal activity, especially against Gram-positive bacteria and yeast. The microbicidal effect of TiO_2_-ZnO nanoparticles varies with each strain. The inhibition halo of the 2Ti:1Zn sample grow up to 14% in most tests, while the 1Ti:2Zn sample produces the smallest halo for *E*. *coli* and *C*. *albicans*; however, for *S*. *aureus*, the halo improves by up to 33%. This phenomenon is attributed to the stoichiometric arrangement capable of inducing oxidative stress. The results show the potential of chitosan films impregnated with TiO_2_-ZnO NPs as functional materials for biomedical applications, especially in the development of dressings with enhanced antimicrobial properties.

## 1. Introduction

According to the World Health Organization (WHO), resistance to antimicrobial agents occurs when drugs lose their ability to eliminate or inhibit the growth of bacteria, viruses, fungi and parasites [1,2]; therefore, the indiscriminate use of drugs constitutes a health risk [3]. In this context, the development of new antimicrobial materials is of great interest.

The use of nanoparticles (NPs) as microbicidal agents has become an innovative and highly efficient strategy compared to conventional methods. They can mitigate the impact of microbial resistance in bacteria and fungi [4], largely because their process facilitates the interaction with biological systems at the molecular level [5]. In addition, their unique properties, such as a high surface-to-volume ratio, durability and their ability to generate reactive oxygen species (ROS), postulate them as materials with potential applications in the field of biomedicine [6].

In particular, the mechanisms of action shown by titanium nanoparticles (TiO_2_ NPs) and zinc nanoparticles (ZnO NPs) include the generation of reactive oxygen species (ROS), alteration of cell membrane integrity and interference with essential metabolic processes in bacteria and fungi [7]. Their chemical stability, biocompatibility and broad antimicrobial spectrum [8] make them viable candidates for biomedical applications [9].

However, material systems with a TiO_2_-ZnO-type core–shell arrangement exhibit improved physical and chemical properties because of the modification of their electron states. Their biomedical applications stand out for their low cytotoxicity, high biocompatibility and remarkable chemical stability [10]. This heterostructured design optimizes the photochemical properties of the nanomaterial, improving its efficiency and providing significant benefits in antimicrobial treatments. For example, Abd El-Fattah et al. [2] developed a hybrid TiO_2_-ZnO nanocomposite that possesses microbial activity against a wide variety of pathogenic microorganisms. The study focused on the incorporation of these nanoparticles into a modified chitosan film, with the aim of evaluating their potential application in food preservation. Veselova et al. [3] synthesized TiO_2_-ZnO nanoparticles and used them to coat cotton textiles by high-power ultrasonic treatment. The results showed that TiO_2_-ZnO NPs exhibit remarkable microbicidal activity against bacteria and fungi. Šebesta et al. [11] evaluated the influence of the use of TiO_2_ and ZnO nanoparticles on agricultural plants under field conditions. The results of the study indicated that the application of these nanomaterials improved plant health and contributed to the mitigation of abiotic and biotic stresses. Dehkordi et al. [12] employed TiO_2_-ZnO nanoparticles mixed in different molar ratios with polyethylene glycol and Portland cement. The study evaluated the mixture as a potential dye decolorizing agent capable of inactivating microorganisms such as *Escherichia coli* and *Streptococcus mutans*.

It should be noted that as the applications of TiO_2_-ZnO nanoparticles increase, it is becoming increasingly important to develop new synthesis methods that are efficient and environmentally sustainable processes. The phytosynthesis has been consolidated as an efficient, ecological and economic alternative for the synthesis of nanoparticles [5,13]. This method is based on the use of a metal solution combined with a plant extract rich in polyphenolic compounds. Since these compounds possess a high antioxidant and reducing capacity, they can promote the nucleation of the metal ions suspended in the aqueous solution, facilitating their bioreduction, stabilization and protection, allowing a controlled synthesis of the nanoparticles [14,15]. For example, Alaizeri et al. [16] synthesized TiO_2_-ZnO nanoparticles using *Senna surrattensis* extract and evaluated their anticancer properties against HCT116 and HuVEC human cancer cells. Mousa et al. [17] used pomegranate and tangerine extract to synthesize TiO_2_-ZnO nanocomposites; subsequently, the resulting nanoparticles were incorporated into a polyvinyl chloride composite membrane, and their photocatalytic activity in human acid degradation was evaluated. The results showed that the process had an efficiency of up to 98.7%.

In particular, the plant *Ruta graveolens* is a medicinal species of the *Rutaceae* family [18], widely recognized for its richness in phytochemicals, including alkaloids, flavonoids and coumarins. Among the most relevant isolated compounds are the acridone alkaloids, as well as furanoacridones such as arborinin and evoxanthin. In addition, several quinolonic alkaloids have been identified, including graveolin [19], all of them with bioactive properties. These compounds may act as reducing agents and metal ion stabilizers, facilitating the synthesis of bimetallic nanoparticles. Also, *Ruta graveolens* exhibits multiple pharmacological benefits, including anti-inflammatory and anticancer effects.

While the synthesis and unique characteristics of nanomaterials play a key role in their application, it is also important to develop viable strategies for their dosage and support. In this context, immobilization of nanoparticles on support materials has become a crucial strategy to enhance the antimicrobial effect and effectively exploit their unique properties [2]. In this regard, the incorporation of TiO_2_-ZnO nanoparticles into chitosan membranes represents a promising alternative, since it optimizes their functional properties [20].

Chitosan is a natural polysaccharide derived from chitin that has aroused great interest as a support material for nanoparticles due to its excellent biocompatibility, biodegradability, and low or no toxicity [21]. Their amino groups constitute the most reactive sites, which facilitate interactions with nanomaterials. This is because the free electron pair of the nitrogen present in the amino group can coordinate with metal ions or with the surface of the nanoparticles [22].

The incorporation of lactic acid into the chitosan membrane can further enhance its properties as a support for nanoparticles by conferring plasticity [23]. Also, lactic acid is a biocompatible and biodegradable compound, which makes it a promising alternative for potential applications in biopharmaceutics [24,25].

The present study aims to phytosynthesize bimetallic TiO_2_-ZnO nanoparticles by using *Ruta graveolens* extract, to support them on a chitosan–lactic acid membrane, and finally, to evaluate their microbicidal activity against *Staphylococcus aureus*, *Escherichia coli* bacteria and *Candida albicans* fungus.

## 2. Materials and Methods

### 2.1. Plant Extract

Native garden leaves of *Ruta graveolens* (Metepec, México, 19° 15′ 01.0” N 99° 34′ 53.5” W), were collected, and washed under running water and allowed to dry at room temperature. Once dried, they were ground and sieved (100 mesh). A total of 10 g of the obtained powder was added to 100 mL of an 80% ethanol–water solution (Sigma-Aldrich, St. Louis, MI, USA). The mixture was kept under constant stirring for 24 h and filtered using a 0.45 µm nitrocellulose membrane (Whatman). The extract was stored in an amber bottle at 4 °C in complete darkness.

### 2.2. Phytosynthesis of TiO_2_-ZnO Nanoparticles

A 0.1 M solution of zinc nitrate (Zn (NO_3_)_2_, Sigma Aldrich) was used as a precursor. A 0.2 M solution of sodium hydroxide (NaOH, J.T. Baker) was added in a 1:1 molar. The obtained zinc hydroxide was decanted and plant extract of *Ruta graveolens* was added in a 1:10 volumetric ratio. The mixture was kept under constant stirring at 60 °C for 1 h.

The same procedure was carried out for the synthesis of titanium dioxide nanoparticles (Anatasa, Sigma Aldrich), mixing in specific molar ratios of the solutions to ensure the ratio of 1 mol TiO_2_ to 1 mol ZnO, 1 mol TiO_2_ to 2 mol ZnO, and 2 mol TiO_2_ to 1 mol ZnO; these mixtures were assigned the following nomenclature: 1Ti:1Zn, 1Ti:2Zn and 2Ti:1Zn, respectively. The resulting TiO_2_-ZnO preparations were heat-treated at 550 °C for 2.5 h and stored in amber vials.

### 2.3. Synthesis of TiO_2_-ZnO NPs-Impregnated Chitosan–Lactic Acid Membranes

The synthesized membranes were obtained as films by the solvent evaporation method. To a solution of chitosan (Sigma-Aldrich, 2–w) and lactic acid (J.T. Baker, Phillipsburg, NJ, USA, 10% volume), TiO_2_-ZnO nanoparticles were added at 0.1 and 0.2–w by volume of the solution. The nanoparticles were dispersed by mechanical stirring, and subsequently with ultrasound. The above dilutions were poured into Petri dishes and dried in a Riossa H-33 oven for 24 h at 60 °C. Finally, membranes were characterized by Fourier Transform Infrared Spectroscopy (FT-IR), Thermogravimetric Analysis (TGA), and Differential Scanning Calorimetry (DSC). Finally, swelling tests were performed with a procedure described in our previous study [26].

### 2.4. Evaluation of Microbicidal Activity

The evaluation of microbicidal activity was carried out using the agar diffusion method. Pathogenic strains of *Escherichia coli* (ATCC 25922), *Staphylococcus aureus* (ATCC 25923) and *Candida albicans* (ATCC 10231) were tested. The concentration of the microorganisms was standardized to 0.5 McFarland, ensuring a population of 1 × 10^8^ CFU mL^−1^ of viable cells.

For the assay, 0.5 mm film disks, previously sterilized by UV irradiation (λ = 254 nm), were used. The prepared plates were incubated at 36 °C for 16 h, after which the formed inhibition halos, expressed in millimeters, were measured. All experiments were performed in duplicate to ensure the reproducibility of the results.

## 3. Results and Discussion

### 3.1. FTIR

**Figure 1:**
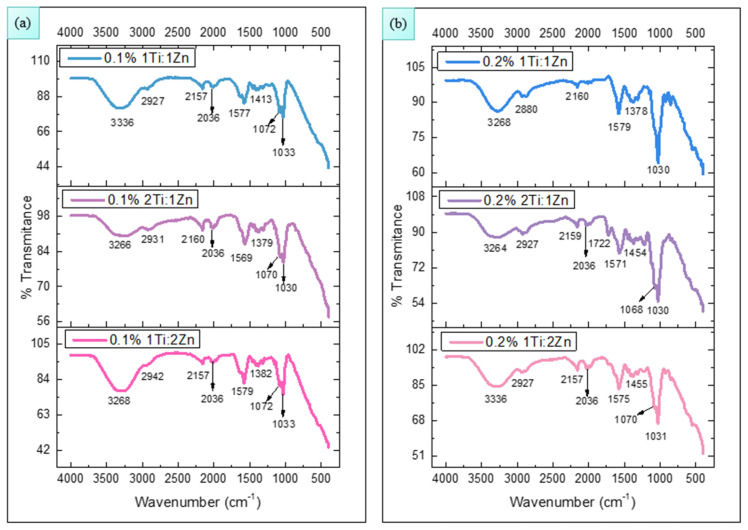
FTIR spectra of chitosan membranes impregnated with TiO_2_-ZnO NPs at 0.1% (**a**) and 0.2% (**b**) *w/v* concentrations.

Fourier Transform Infrared Spectroscopy (FTIR) analysis was performed on the Jasco FT/IR 4X View spectrophotometer (Thermo Fisher Sci., Tokyo, Japan), configured at a resolution of 4 cm^−1^ and 32 scans in the spectral range from 4000 to 400 cm^−1^. Figure 1a,b represent the results of FTIR analysis of membranes impregnated with TiO_2_-ZnO nanoparticles (1Ti:1Zn, 1Ti:2Zn, 2Ti:1Zn) at concentrations of 0.1 and 0.2% *w*/*v*, respectively.

Around 3300 cm^−1^, the stretching vibrations of amine (-NH_2_) and hydroxyl (-OH) groups are located [27], while the peak located at 2927 cm^−1^ is associated with the stretching vibrations of alkali (-CH) groups from chitosan and in lactic acid [28].

During membrane synthesis, although an ultrasonic sonicator is employed to reduce the quantity of bubbles containing ambient air in the membranes, the presence of the peaks located at 2160 and 2036 cm^–1^ could indicate that CO_2_ is still present in the sample.

At 1579 cm^−1^ the stress vibrations of the primary amine N-H groups (-NH_2_) of chitosan are observed [2]. The slight shift in this peak in the samples is due to the chitosan interacting with lactic acid and protonating the amine groups (forming -NH_3_^+^) [29]; there may also be a contribution from the asymmetric vibration of the carboxylate group (COO-) of lactic acid by its interactions with TiO_2_-ZnO NPs [30].

The peak located at 1455 cm^−1^ is generally associated with the stress vibrations of the C-H groups (mainly CH_2_ and CH_3_) of chitosan and lactic acid. In the absorption range of 1070 and 1030 cm^−1^, the characteristic peaks of the stretching vibrations of the C-O groups (of the primary and secondary alcohols) and C-O-C of the glycoside bridge are found.

By FT-IR, it can be observed in all the analyzed samples that the intensity of these absorption peaks decreases or increases depending on the concentration of TiO_2_-ZnO NPs present in the membrane [8,31]. This behavior could indicate the consolidation of crosslinking between chitosan and nanoparticles impregnated in the membrane matrix.

### 3.2. TGA-DT

**Figure 2:**
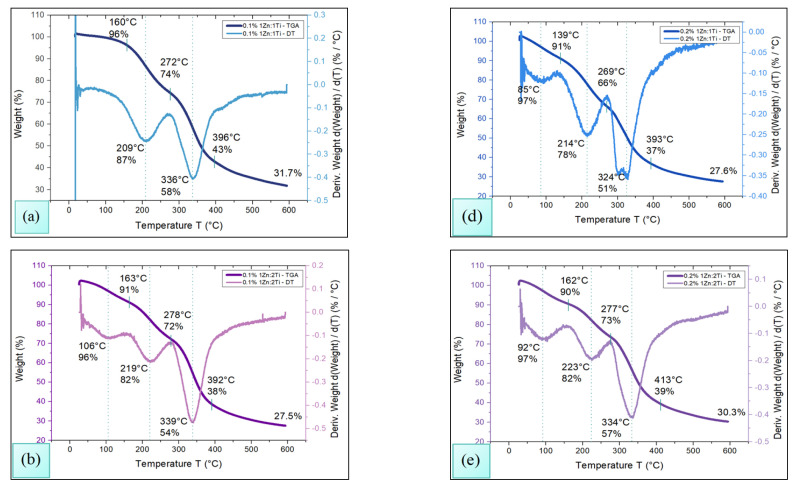

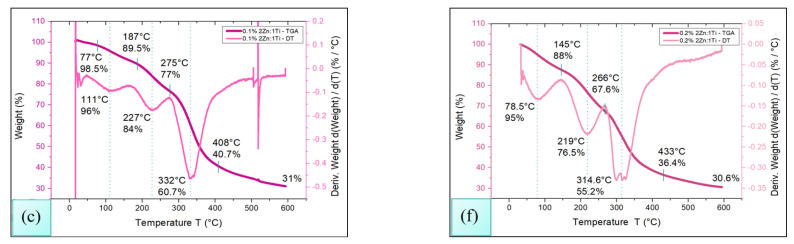
TGA analysis for chitosan membranes impregnated with TiO_2_-ZnO NPs at concentrations of 0.1% (**a**) 1Ti:1Zn, (**b**) 2Ti:1Zn, (**c**) 1Ti:2Zn, and 0.2% *w/v* (**d**) 1Ti:1Zn, (**e**) 2Ti:1Zn, (**f**) 1Ti:2Zn.

Thermogravimetric Analysis (TGA) of chitosan membranes impregnated with TiO_2_-ZnO nanoparticles was performed using the TA Instrument Discovery TGA 550 (Waters, New Castle, DE) under a controlled atmosphere of N_2_. The procedure consisted of heating the samples from an initial temperature of 30 up to 600 °C, at a rate of 10 °C/min, using a platinum sample holder. The results of the study are shown in Figure 2.

The gradual mass loss allows the identification of at least three important zones, related to the decomposition of the different compounds that are part of the polymer. The first zone, located in the temperature range T ≤ 150 °C, is associated with the loss of moisture from the membrane (87.7 ± 11.8 °C) and the boiling of the zinc hydroxide (91.5 °C ± 14.2 °C) [32].

The second zone is in the range of 150 °C ≤ T ≤ 350 °C, where chitosan depolymerizes (218.5 °C ± 6.4 °C) causing considerable weight loss [33], glycerol evaporates (272.8 °C ± 4.7 °C), and lactic acid dehydration takes place (159.3 °C ± 16.7 °C) which probably results in the formation of the lactide dimer [23,34] which eventually evaporates (329.9 °C ± 9 °C).

Finally, in the third zone, ranging from 350 °C ≤ T ≤ 500 °C, the pyrolysis of chitosan (405.8 °C ± 15.8 °C) occurs, as does the combustion and decomposition of all the organic matter present in the sample, the TiO_2_-ZnO NPs and other residues being the most prominent.

### 3.3. DSC

**Figure 3:**
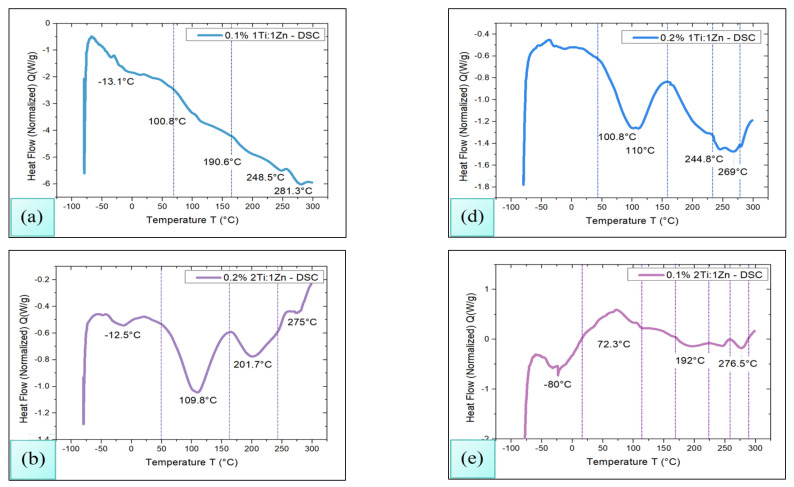

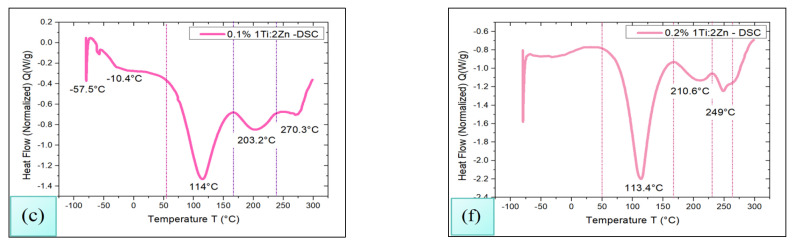
DSC analysis for chitosan membranes impregnated with TiO_2_-ZnO NPs at concentrations of 0.1% (**a**) 1Ti:1Zn, (**b**) 2Ti:1Zn, (**c**) 1Ti:2Zn, and 0.2% *w/v* (**d**) 1Ti:1Zn, (**e**) 2Ti:1Zn, (**f**) 1Ti:2Zn.

To determine the glass transition temperature (Tg) as well as the decomposition temperature of the samples, Differential Scanning Calorimetry (Waters, New Castle, DE, USA) analysis was performed using TA Instrument Discovery DSC 250 equipment. The procedure consisted of heating the samples from room temperature to 300 °C in a sealed aluminum capsule, with a nitrogen flow rate of 60 mL/min and a heating rate of 10 °C/min. The results are shown in Figure 3. At first instance, it can be observed that endothermic processes at low temperatures are identified for all samples, alluding to the possible Tg of the chitosan, in some amorphous phase of the polymer or in the loss moisture from the membrane [32].

On the other hand, the analyses reveal the presence of exothermic peaks in at least three important zones. The first zone is located around 110 °C in the TGA-DT analysis; it was also possible to observe the loss of the volatile material at this temperature, as the DSC analysis make its presence evident, which may indicate that at the temperature of 110 °C ± 2 °C at least three relevant processes develop: (i) crosslinking induced by the interaction between the TiO_2_–ZnO nanoparticles and the chitosan of the membrane [35]; (ii) dehydration of lactic acid with the formation of lactide, followed by its possible reaction with the metal oxides present to give rise to metal lactates [34]; and (iii) crystallization of chitosan, which involves a structural reorganization of its polymer chains, modifying its mechanical properties and increasing its solubility [24].

It is noteworthy that the TGA-DT analysis integrates the information on the degradation process of chitosan, which begins to depolymerize around 210 °C ± 5 °C, revealing that the polymeric chains of chitosan were split into smaller fragments. This phenomenon is probably caused by the breakdown of glycosidic bonds of chitosan; in addition, the lactic acid had also begun to degrade [36].

Finally, DSC analysis shows that at 260 °C ± 6.6 °C the beginning of the thermal degradation of chitosan develops [37]; this phenomenon is remarkable in TGA-DT analysis. It can also be concluded that the decomposition of the polymetric matrix has been carried out and only TiO_2_-ZnO NPs in some of their possible amorphous phases remain as remnants.

### 3.4. Swelling Tests

**Figure 4:**
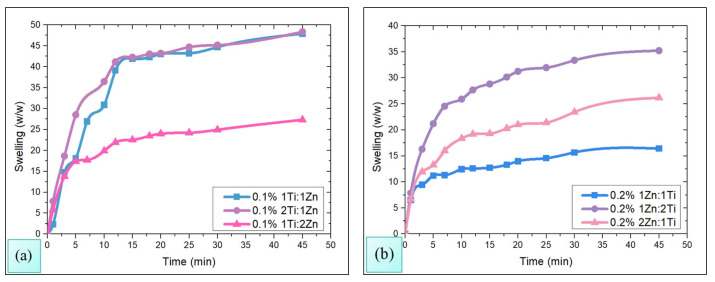
Swelling tests of chitosan membranes impregnated with TiO_2_-ZnO NPs at concentrations of 0.1% (**a**) and 0.2% (**b**) *w*/*v*.

The results of the swelling tests of chitosan membranes impregnated with TiO_2_-ZnO NPs are shown in Figure 4a,b. In Figure 4a, it can be observed that at lower concentrations of nanoparticles (0.1% *w*/*v*), membranes tend to have a higher degree of swelling, and as the concentration of TiO_2_-ZnO NPs increases (0.2% *w*/*v*, Figure 4b), the degree of membrane swelling becomes limited.

This behavior can be associated with the fact that the membranes impregnated with TiO_2_-ZnO, in a stoichiometric ratio 1Ti:1Zn and 2Ti:1Zn, still have available spaces in the chitosan matrix capable of absorbing water, thus exhibiting a higher degree of swelling up to 45% *w*/*w*. Meanwhile, the 1Ti:1Zn and 2Ti:1Zn membranes experienced a drastic reduction in the degree of swelling expanding only by 15% and 35% *w*/*w* respectively, thus indicating a greater inherent limitation in their swelling capacity, possibly due to a more restrictive interaction with chitosan [38].

On the other hand, the 1Ti:2Zn membrane maintained the same degree of swelling for the two concentrations of employed nanoparticles; this behavior suggests that the membranes are already significantly limited at a lower concentration. Therefore, the stoichiometric composition of the nanoparticles plays a crucial role in modulating the membrane swelling, especially at higher concentrations where the effect of nanoparticles as limiting agents becomes more noticeable.

### 3.5. Microbicidal Test Evaluation

**Figure 5:**
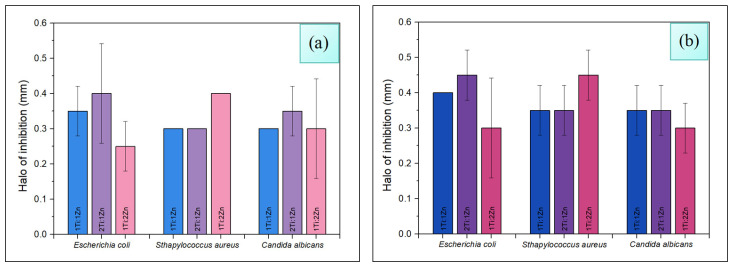
Measurement in mm of the inhibition halos generated by chitosan membranes impregnated with TiO_2_-ZnO NPs at concentrations of 0.1% (**a**) and 0.2% (**b**) *w*/*v*.

The results of the antimicrobial evaluation of chitosan membranes, impregnated with TiO_2_-ZnO nanoparticles against *Escherichia coli*, *Staphylococcus aureus*, and *Candida albicans* microorganisms using the disk diffusion method (Kirby–Bauer), are presented in Figure 5.

TiO_2_-ZnO nanoparticles with a stoichiometric ratio 2Ti:1Zn and 1Ti:2Zn at concentrations of 0.2–*w*/*v* exhibited higher antimicrobial efficiency against *Escherichia coli* and *Staphylococcus aureus* [39]. This behavior could be attributed to the higher content of titanium dioxide and zinc oxide, and their affinity for bacterial cells [4]. Such affinity allows a more effective interaction with microbial cells, facilitating the disruption of cytoplasmic membrane integrity [8]. This effect is enhanced by the generation of reactive oxygen species (ROS), inducing significant structural damage to both bacterial and fungal cell walls [8,40], triggering the process of cell death [41].

On the other hand, the 1Ti:1Zn arrangement shows consistent microbicidal action in all cases. Although it is known that the toxicity of TiO_2_ is mainly related to its physical properties [3], rather than to its chemical structure [4], the decreased performance of 2Ti:1Zn and 1Ti:2Zn could be due to the higher surface heterogeneity of TiO_2_ [39,42]. This irregularity may interfere with proper interfacial charge separation, limiting the efficient generation of ROS, and thus reducing its microbicidal capacity.

**Figure 6:**
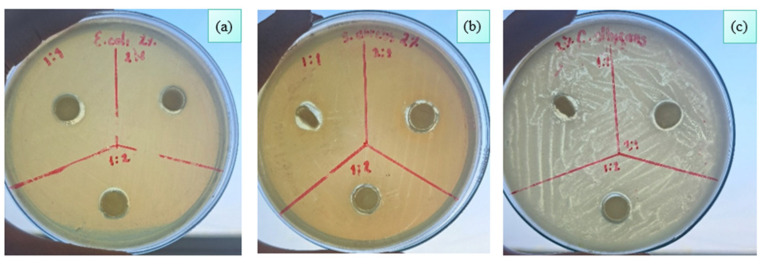
Antimicrobial effect for (**a**) *Escherichia coli*, (**b**) *Staphylococcus aureus* and (**c**) *Candida albicans* against chitosan membranes impregnated with 0.2% *w/v* TiO_2_-ZnO NPs.

The difference in antimicrobial and antifungal activity observed in TiO_2_-ZnO nanoparticles can also be attributed to the structural characteristics of the microorganism evaluated (Figure 6). *Escherichia coli* [41], as a Gram-negative bacterium, presents a cell envelope composed of a semipermeable lipid bilayer, reinforced by an inner plasma membrane and an outer membrane, separated by a periplasmic space containing a thin peptidoglycan layer [43]. This complex configuration may hinder the penetration of nanoparticles and limit their interaction with intracellular components.

Instead, *Staphylococcus aureus* (Gram-positive) and *Candida albicans* (fungus), although differing in nature, possess less complex cell membranes, with lipid bilayers acting as selective barriers [44]. In these cases, TiO_2_-ZnO NPs seem to exert a more effective activation. In the case of *Candida albicans*, induced damage to the cell wall can generate an osmotic imbalance [40], leading to membrane rupture and the release of cytoplasmic contents, which ultimately results in cell death [7].

The use of plant extracts, as an environmentally friendly method, produces a film that coats the metal nanoparticles and helps them stabilize [10]. This film may contain bioactive compounds such as pyrazine, which forms hybrids with thiazole and exhibits microbicidal properties. These could contribute to the same effect as the metallic nanoparticles, resulting in a material with minimal potential toxicological risk [45].

In all cases, the integrity of the cell wall constitutes a vital component for the vitality of the microorganism and its alteration by the action of nanoparticles represents a key mechanism in the observed antimicrobial effectiveness [43]. This principle is consistent with recent findings on antiviral polymeric nanocoatings, which inactivate variants of SARS-CoV-2 by disrupting the viral envelope and degrading RNA, thus expanding the concept of pathogen inactivation through surfaces functionalized with nanomaterials [46].

## 4. Conclusions

The present study confirms that the phytosynthesis process is a sustainable, efficient and low-cost alternative for obtaining bimetallic TiO_2_-ZnO nanoparticles with antimicrobial properties. The incorporation of TiO_2_-ZnO NPs in a chitosan matrix improved the mechanical properties and microbicidal activity of the material, even at low concentrations.

FT-IR analysis evidenced the correct impregnation of TiO_2_-ZnO NPs in the polymeric matrix, while microbiological assays indicated their release capacity and microbicidal action. In turn, thermal analysis showed that the membranes are stable against temperature variations, and swelling tests confirmed their structural resistance, as they did not exhibit deformations.

The microbicidal effect of TiO_2_-ZnO nanoparticles varies with each strain. For *E*. *coli* and *C*. *albicans*, the 2Ti:1Zn sample improves by 14% compared to 1Ti:1Zn, while for the *S*. *aureus* sample, the 1Ti:2Zn sample improves by up to 33% compared to the other two. As the amount of nanocomposite used increases, the behavior is similar for *E*. *coli* and *S*. *aureus*, but not for *C*. *albicans*, where the effect of the 1Ti:1Zn and 2Ti:1Zn samples is similar.

Overall, the results highlight the potential of the chitosan TiO_2_-ZnO NPs system as a functional material with potential applications as a microbicidal agent, with proven antimicrobial efficacy against *Escherichia coli*, *Staphylococcus aureus,* and *Candida albicans*.

## Data Availability

All data generated or analyzed during this study are included in this manuscript.
